# Supplementary material for: How predictive of SARS-CoV-2 infection are clinical characteristics at presentation among individuals with COVID-like symptoms treated at the Mexican Institute of Social Security
Source: PLoS One. 2023 Dec 21;18(12):e0296320. doi: 10.1371/journal.pone.0296320 (PMC10735012; doi:10.1371/journal.pone.0296320)
Supplement: Attachment — Submitted filename: Response to reviewers.docx [file pone.0296320.s001.docx]

| Comment | Answer/action |
| --- | --- |
| Reviewer 1 |  |
| As the title suggests, the intent is to show how the pandemic evolved over time. Is it perhaps more informative that a comparison within result status be performed? For example, did the prevalence of fever among COVID-positive change between the 3 waves? Some of the most common symptoms do vary between test result and age group, however these prevalences appear to be relatively similar across waves. This is more apparent in Figure 1 as compared to Table 1. It is interesting, particularly among ambulatory, that some of the biggest prevalence changes occur among the negatives? In that regard, are the difference changes listed in Table 1 driven more by evolution of COVID-negative or COVID-positive? | Thanks for your comment. As reviewer 2 highlighted, our aim is to evaluate the predictive power of signs and symptoms to identify COVID-positive cases and severe cases. While we acknowledge that a comparison of prevalence by other characteristics --such as age-- would be interesting, that is not the scope of this paper and will be the subject of future work. As for the changes in prevalence by wave, we report those changing prevalences in table 1. Does that address the concern? |
| Figure 1 is cumbersome to view | We decided to remove figure 1 as it is not central to the argument and we agree with the reviewer that it is difficult to interpret. |
| The authors describe how clinical presentation varies by patient age as well as by location type (i.e., ambulatory vs. hospitalized). It would be worth noting whether the distribution of these two variables changed between Waves. Presumably they do but it may help the reader better understand what may be driving changes over time. Relatedly, there is no formal definition of ambulatory vs. hospitalized. This may be especially relevant if testing occurred in an ambulatory setting but then there was an escalation of care. | As we mention above (and in response to reviewer 2’s suggestions) we have tried to focus the paper more clearly on the central question as described above – predictive power – rather than a description of the evolution of the pandemic in Mexico. |
| As Figure 1 suggests, the ROC values may show greater discerning ability for select age groups, rather than the overall population. Was there an examination of ROC by select age groups? | No we had not included that initially, but we have now included Table 3 in response to this suggestion. |
| The distinction between suspect and severe are somewhat blurred since dyspnea and chest pain are included in both definitions. | We acknowledge this, but we are using the official definitions in Mexico. In their definition, all of the severe cases are also suspect cases. |
| It’s unclear why some of the prevalences in Table 2 are discrepant from Table 1. For example, the lowest proportion for headache in Table #1 is 75.3% but the proportions for Wave 1 and Wave 2 are roughly 65%. | It was a mistake, thanks for noticing it. We were using the prevalence including not-tested individuals in table 1. We corrected the table for the revised version. |
| Line 76 states that only suspected [or severe] cases were included in the analysis. Consequently, examining the relationship between suspected case status and positive/negative result (Line 138) doesn’t seem clear | We included suspected cases who also received a test and who were thus either positive or negative. Everyone was a suspected case and therefore we can’t say anything about the relationship between being suspected and the probability of being positive. That would only be possible if we also had test results from patients who were not suspected of having COVID – but we don’t. |
| Referencing the IMSS is helpful but the authors should consider adding an additional sentence describing how symptom data are collected. This will help the reader better understand the accuracy of select symptoms that may be hard to document in particularly young patients. | We added additional details on this. |
| There are typos that need to be corrected. | We have tried to correct the typos. |
| Reviewer 2 |  |
| I would recommend to the authors to improve the manuscript by clarifying its main objective and message and by rewording the title accordingly. | We amended the objective in the abstract and intro, as well as the title, per reviewer recommendation. |
| Lines 62-63: the last sentence should be removed and placed in the discussion and/or in the conclusion instead | Agreed. Removed. |
| Methodology: the methods should more detailed. There is no mention of how missing values were treated. This should be described here. Table 1: Add N for each category + add p-values | We included details on missing values.  Bottom row of table 1 reports the n for each column. As we are using data from a complete set of individuals, that is, not a sample of a larger population, there is no sampling bias so differences in proportions are real and not subject to random sampling error (that would be reflected in a p-value). It is analogous to my saying that I have one son and two daughters. I don’t include an estimate of uncertainty of the estimate related to the sample size and the variance because it is a 100% sample of my children. If I were to estimate the proportion of male and female children in the Bay Area based on the sample of three children then I would need to report the p-value. Our results should not be interpreted as representative of the entire Mexican population because the patients treated at the IMSS are not similar to those treated in other health systems.  Additional details on the methods were included. |
| Discussion: Add a short summary of the findings of the study and an analysis of its strength and weaknesses. | We revised the discussion accordingly with the reviewer suggestions. |
| Conclusions: there is no conclusion in the main text | We added conclusions |
| There are some typing errors that should be corrected (ex line 102) | We corrected the errors we were able to find. |
| Line 107: Authors wrote : “Figure 1 and 2….” but there is only one figure in the document (Figure 1). On this figure, the group age are not readable. The police should be increased. | We removed the figure and the references to it as described in the response to Reviewer 1. |
