# Supplementary material for: How predictive of SARS-CoV-2 infection are clinical characteristics at presentation among individuals with COVID-like symptoms treated at the Mexican Institute of Social Security
Source: PLoS One. 2023 Dec 21;18(12):e0296320. doi: 10.1371/journal.pone.0296320 (PMC10735012; doi:10.1371/journal.pone.0296320)
Supplement: Attachment — Submitted filename: Answers to reviewers 09Jun2023.docx [file pone.0296320.s002.docx]

| Coment | Action |
| --- | --- |
| Additional detail on IMSS has been added. However, I believe the paper could be strengthened further by including additional descriptions. For example, are there any validation or reliability statistic that IMSS has calculated? Something to assure the reader that medical data were abstracted in a reliable manner. This become more relevant, perhaps, when trying to understand how accurately some of these symptoms could be assessed among infants (e.g., headache, anosmia). | We understand the reviewer's concern; However, the SINOLAVE data was validated and recorded as described in the manuscript by the staff responsible for the epidemiological surveillance. |
| A level of precision is needed, be it confidence intervals (preferable) or p-values. | Confidence intervals were added to the tables 2&3. Nevertheless, as the CIs are extremely tight, it is not clear the value added. For table 1, we added whether the difference between positives and negatives was significant, as adding CIs here could make the table unreadable. |
| Relatedly, there are no sample sizes in Table 3 to understand the frequency contribution of the 3 age groups. | The number of observations was added to Table 3 |
| Analysis describes a subgroup-analysis to compare positive non-severe and positive severe, but the reviewer was unable to find those results. | We deleted this sentence; the idea was to refer to the presented comparison between suspect and severe cases as defined in the guidelines. |
| Why is anosmia included in the “main 6” but it’s not listed in Table 1? | Included now, as well as dysgeusia |
