# Supplementary material for: How predictive of SARS-CoV-2 infection are clinical characteristics at presentation among individuals with COVID-like symptoms treated at the Mexican Institute of Social Security
Source: PLoS One. 2023 Dec 21;18(12):e0296320. doi: 10.1371/journal.pone.0296320 (PMC10735012; doi:10.1371/journal.pone.0296320)
Supplement: Attachment — Submitted filename: Answers dec2023.docx [file pone.0296320.s003.docx]

| Comment | Answer |
| --- | --- |
| Line 6: Authors should use numbers to denote authors names and not symbols | Done |
| Line 59: Authors should amend COVID to COVID-19 | Corrected |
| Line 73: Authors should replace the statement "to be nadir in cases" with a more appropriate sentence | Edited |
| Line 84: Authors should indicate the software used for the analysis | It was already mentioned below, but added here as well. |
| Line 98: Authors should amend COVID-19,, to "COVID-19" | We could not find the referred text |
| Line 102: Authors should correct the sentence "SARS-COV-2aespecially" and remove the bracket at the end of the sentence | We could not find the referred text |
| Line 105: Authors should include the total numbers for each symptom and wave observed. This will help improve understanding of the data | Included |
| Line 112: Authors should correct "high lev" | We could not find the referred text |
| Line 136: Authors should remove "in" and correct the sentence. | We could not find the referred text |
| Line 151: Authors should explain the full meaning of "main" under the table. The various symptom combinations should be indicated | Added |
| Line 154: Authors should review literature on other studies which have describe the ROCs of these symptoms and other they compare to this current findings. The discussion needs more through review | We edited the discussion, and added new references. |
